# Reply letter to the commentary on “Clinical efficacy and biomarker analysis of neoadjuvant camrelizumab plus chemotherapy for early-stage triple-negative breast cancer: a experimental single-arm phase II clinical trial pilot study”

**DOI:** 10.1097/JS9.0000000000002162

**Published:** 2024-12-20

**Authors:** Xiangjing Meng, Xingchen Meng, Yuechen Wang, Ning Yan, Chunhui Zheng

**Affiliations:** a Toxicology Research Center, Shandong Academy of Occupational Health and Occupational Medicine, Shandong First Medical University & Shandong Academy of Medical Sciences, Jinan, Shandong, China; b Breast Disease Department, Weifang People’s Hospital, The First Affiliated Hospital of Shandong Second Medical University, Weifang, Shandong, China

*Dear Editor,*

We wish to thank Zhang *et al* for their constructive comments on our study “Clinical efficacy and biomarker analysis of neoadjuvant camrelizumab plus chemotherapy for early-stage triple-negative breast cancer: a experimental single-arm phase II clinical trial pilot study”^[1,2]^. In this reply letter, we shall attempt to answer all these questions. We have studied each comment carefully and our responses are as following:

Firstly, Zhang *et al* pointed out that the main limitation of this study is the lack of a randomized control group. We also appreciate the acknowledgment of the central role of randomized control group in oncology. However, the single-arm phase II clinical trial is primarily used to initially evaluate the efficacy and safety of a new drug, and this trial design is characterized by having only treatment group and no control group. Importantly, the advantage of the single-arm phase II clinical trial pilot study is its ability to quickly and efficiently evaluate the efficacy of a new treatment strategy in a specific patient population.

Secondly, the small sample size is highlighted as another limitation. This is because triple-negative breast cancer approximately accounting for 15% of invasive breast cancers^[3]^, as well as this was a single-center prospective study. Besides, we enhance the credibility of our findings through animal validation and data sharing.

Thirdly, as Zhang *et al* mentioned, to determine the optimal course of treatment, we are conducting a multicenter, real-world, large-sample size study. Furthermore, in neoadjuvant therapy, there are still multiple issues that need to be resolved, such as the rates of breast conservation and axillary preservation. Further exploration is required through multicenter, large-sample, prospective studies.

## Data Availability

Not applicable.
